# Is it sufficient to evaluate COVID‐19 infected critically ill patients only in terms of VTE risk factors? What about disease severity?

**DOI:** 10.1111/crj.13520

**Published:** 2022-07-05

**Authors:** Fatma Yildirim, Mehmet Yildirim

**Affiliations:** ^1^ Department of Pulmonary Diseases, COVID Intensive Care Unit University of Health Sciences, Diskapi Yildirim Beyazit Research and Education Hospital Ankara Turkey; ^2^ Department of Internal Medicine, COVID Intensive Care Unit University of Health Sciences, Diskapi Yildirim Beyazit Research and Education Hospital Ankara Turkey

Dear Editor,

We read with great interest the study by Wang et al.^1^ which was published online and being ahead of print in the *Clinical Respiratory Journal*. At the beginning of the corona virus disease 2019 (COVID‐19) pandemia in their single‐centre retrospective, observational study, they retrospectively analysed 138 COVID‐19 patients and researched the factors for in‐hospital venous thromboembolism (VTE) and bleeding. They found that 16.7% of patients with COVID‐19 had high risk for VTE according to Pauda Prediction Score; 6.5% of patients had high risk of bleeding for VTE prophylaxis according to Improve Prediction Score. They identified thrombotic events in four patients (2.9%), all of them had deep vein thrombosis (DVT). They found the incidence of VTE as 20% and one major haemorrhage was happened in critically ill patients during VTE treatment. They defined critically ill patients as those admitted to intensive care unit (ICU) who required mechanical ventilation or had a fraction of inspired oxygen (FIO_2_) of at least 60% or more. They used lower extremity compression ultrasound to detect DVT for all critically ill patients with a high risk of VTE and high level of D‐dimer. If ultrasound was positive, patients underwent computed tomography pulmonary angiogram.^1^ This is an important issue, but there are some practical questions to be answered for a proper clinical extrapolation.

First, COVID‐19 leads coagulation disorders that are triggered by increased level of procoagulant substances. Sepsis‐induced coagulopathy (SIC) and disseminated intravascular coagulation (DIC) are the main causes of coagulation associated mortality in critically ill patients. The SIC criteria established by the International Society on Thrombosis and Haemostasis (ISTH) is often used to guide anticoagulant therapy in hospitalised patients.^2^ Nevertheless, DVT occurs in majority of patients with critically ill COVID‐19. COVID‐19 is an acute viral infection which primarily affects respiratory system and if it progresses to acute respiratory distress syndrome (ARDS) and leads other organ failures, as well. Although Wang et al.^1^ used Padua Prediction Score and found 16.7% of the patients were at high risk for VTE, VTE was determined in 20% of the patients. According to Padua Prediction Score, patients with chronic conditions such as malignancy, reduced mobility, and thrombophilia get higher risk scores whereas common disorders during COVID‐19 course such as heart and respiratory failure, acute myocardial infarction, or ischaemic stroke result in minor score increase. This is the most likely reason of low prediction rate which is reported in the previous study.^1^ Moreover, apart from thromboembolic situation such as DVT, in patients with COVID‐19, pulmonary inflammation may lead susceptibility to embolism.^2^ Therefore, not only suspicion for DVT, presence of ARDS, elevated D‐dimer levels, or worsening in oxygenation should make clinicians consider pulmonary thrombosis and perform chest imaging.

Second, it has shown by many studies that severity of COVID‐19 is a risk factor for VTE and elevated ferritin, C‐reactive protein, D‐dimer, and interleukin‐6 levels are associated with thromboembolic events and poor prognosis.^3^ In the previous study by Wang et al.,^1^ there is not enough data on duration and severity of disease. We believe that considering these factors together with Padua Prediction Score provides better prediction rates for VTE.

Third, to determine COVID‐19 associated thromboembolism, Sequential Organ Failure Assessment (SOFA) score that is one of the disease severity and organ failure scores has been evaluated by some previous studies.^4^, ^5^ These studies suggest that in critically ill COVID‐19 patients, higher SOFA scores are associated with higher DVT frequency, D‐dimer levels, duration of disease, hypoalbuminemia, and duration of disease.^4^ Other previous study^5^ reported that higher ISTH SIC scores which include SOFA score, prothrombin time–international normalised ratio (PT‐INR), and platelet levels are associated with higher mortality and patients with ISTH SIC score higher than 3 had 3.05‐fold increased mortality risk. Both haemodynamic situation and arterial oxygen partial pressure to fractional inspired oxygen ratio (PaO_2_/FIO_2_) are components of SOFA score. PaO_2_/FIO_2_ ratio is a main determinator of ARDS severity and also reflects the severity of COVID‐19.

Finally, we believe that further prospective clinical trials need to develop an effectual risk prediction score including disease severity to determine VTE earlier in COVID‐19 patients.

## CONFLICT OF INTEREST

The authors declare that the letter was conducted in the absence of any commercial or financial relationships that could be construed as a potential conflict of interest.

## AUTHOR CONTRIBUTIONS

All authors contributed equally to the letter.

## Data Availability

Data sharing is not applicable to this article as no new data were created or analyzed in this study.
